# Structure–Property Relationship in Ultra-Thin Copper Foils: From Nanotwinned to Fine-Grained Microstructures

**DOI:** 10.3390/ma19010036

**Published:** 2025-12-21

**Authors:** Fu-Chian Chen, Dinh-Phuc Tran, Chih Chen

**Affiliations:** Department of Materials Science and Engineering, National Yang Ming Chiao Tung University, Hsinchu 300093, Taiwan; fuchianchen.mse@gmail.com (F.-C.C.); trandinhphuc1508@gmail.com (D.-P.T.)

**Keywords:** Cu foil, mechanical properties, nanotwinned Cu, columnar grain Cu, fine grained Cu

## Abstract

This study systematically investigates the thickness-dependent mechanical properties of electroplated copper foils with fine-grained (FG-Cu) and columnar nanotwinned (NT-Cu) microstructures. Tensile testing across a thickness range of 5–30 μm revealed that NT-Cu exhibits superior mechanical stability, with significantly lower reductions in both ultimate tensile strength (UTS) and yield strength (YS) compared to FG-Cu. The UTS of the 30 μm thick FG-Cu foil was measured at 651 MPa, increasing to 792 MPa at a thickness of 5 μm. In contrast, the UTS of NT-Cu foils only rose from 624 MPa at 30 μm to 663 MPa at 5 μm. A similar trend was observed for the YS. Microstructural analysis confirmed that NT-Cu maintains a stable columnar grain structure with minimal grain growth, contributing to its resistance to thickness-induced strength loss. These findings highlight NT-Cu as a promising candidate for applications requiring consistent mechanical performance across varying foil thicknesses.

## 1. Introduction

Copper foils play a critical role in a wide range of modern technologies, including flexible electronics [1], microelectronics [2], printed circuit boards [3], and energy storage systems [4]. Their excellent electrical and thermal conductivity, combined with favorable mechanical properties, make them ideal for use as interconnects in advanced packaging [5], flexible circuits in wearable devices [6,7], current collectors in lithium-ion batteries (LIBs) [8]. As applications diversify, the required thickness of copper foils spans a broad range—from ultra-thin foils (<10 μm) for foldable displays [9,10] and stretchable electronics [11,12], to thicker variants (>20 μm) for structural interconnects and battery components. This wide thickness spectrum presents a key engineering challenge: ensuring that the mechanical properties of copper foils remain stable across different thicknesses to support process compatibility, structural reliability, and long-term performance.

However, the mechanical behavior of copper foils is highly dependent on both their thickness and microstructure. When the foil becomes thinner [13], size-dependent deformation mechanisms such as grain boundary sliding [14], and surface/interface effects [15,16] become increasingly dominant, often leading to significant variations in strength and ductility. To meet industrial requirements, copper foils must exhibit mechanical robustness, defined as minimal variation in tensile or yield strength with changing thickness. Achieving such consistency is particularly challenging with conventional polycrystalline copper.

To enhance the mechanical performance of copper, several classical strengthening mechanisms are commonly employed: grain refinement [17], work hardening [18], precipitation hardening [19], and solid solution strengthening [20]. Nanotwin strengthening has recently been recognized as a fifth and distinct mechanism. Nanotwinned copper (NT-Cu), characterized by high-density nanoscale twin boundaries, offers an attractive balance of high tensile strength [21], ductility [22,23], thermal stability [24,25], and low electrical resistivity [26,27]. NT-Cu can be fabricated by magnetron sputtering or electroplating. Although sputtering produces highly oriented, dense twin structures, its low deposition rate limits scalability. In contrast, electroplated NT-Cu is more compatible with mass production processes.

Although a few studies have examined the effect of thickness on NT-Cu, the available literature presents several unresolved gaps. First, most prior work focuses exclusively on nanotwinned structures or on a single foil thickness, preventing direct evaluation of how thickness modifies the relative strengthening contributions of grain boundaries and twin boundaries. Second, the thickness ranges reported in earlier studies are often narrow or discontinuous [28,29], and results are further complicated by variations in the electroplating chemistry [29], waveform [30], or deposition conditions [31], making it difficult to isolate the true thickness effect. Third, no study to date has systematically compared FG-Cu and NT-Cu foils prepared under identical electroplating conditions across a continuous thickness range, nor quantified how twin density, misorientation distribution, or texture evolve with thickness. These knowledge gaps limit the current understanding of the underlying size-dependent deformation mechanisms governing ultra-thin Cu foils.

To address these limitations, this study provides a systematic investigation of the mechanical properties of electroplated FG-Cu and NT-Cu foils with thicknesses ranging from 5 to 30 μm, all produced under identical electrolyte composition and deposition conditions. By combining uniaxial tensile testing, EBSD-based microstructural characterization, and Hall–Petch analysis, we directly compare the strengthening behavior of FG-Cu and NT-Cu and quantify how microstructure evolves with foil thickness. This approach eliminates processing-related variability and enables a clear assessment of the thickness effect in both microstructural systems. The objective of this work is therefore to establish the thickness–property relationships for FG-Cu and NT-Cu foils within a controlled electroplating system. The results provide new insight into the deformation mechanisms of ultra-thin Cu foils and offer practical guidance for the design of high-performance metallic films for microelectronic and flexible device applications

## 2. Experimental

**Figure 1:**
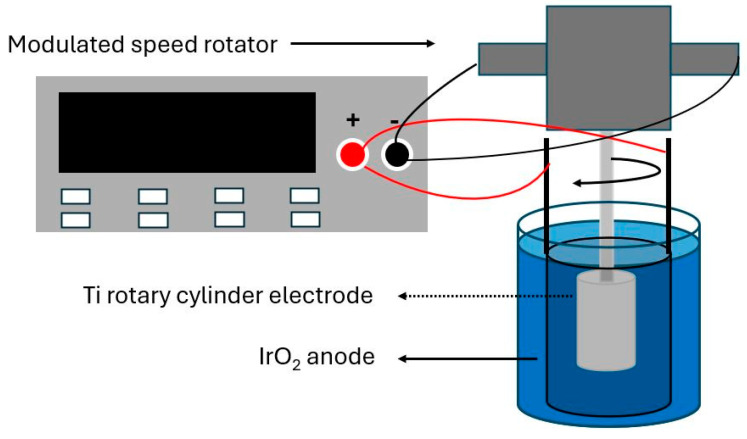
Schematic of rotary electroplating system.

In this study, Cu foils were produced using a rotary electroplating system, illustrated in Figure 1. A titanium (Ti) cylinder was employed as the cathode substrate due to its weak interfacial adhesion with Cu, which allowed for easy detachment of the plated Cu foil after deposition. The Ti cathode was mounted onto a variable-speed rotary mechanism and operated at a fixed rotational speed of 300 revolutions per minute (rpm). Surrounding the cathode, a hollow cylindrical anode made of titanium coated with iridium dioxide was positioned concentrically. The electrolyte bath was composed of 50 g/L copper (II) sulfate pentahydrate (CuSO_4_·5H_2_O), 40 ppm chloride ions (Cl^−^), and 100 g/L concentrated sulfuric acid (96% H_2_SO_4_), all dissolved in 1 L of deionized water.

Two different additive combinations were used in this experiment: DP112 + DP114 and DP101 additive (Chemleader Inc., Hsinchu, Taiwan), which enable the electroplating solution to produce copper foils with fine-grained and columnar microstructures, respectively.

Various electroplating times were applied in this study. The electroplating time varies from 140 s to 860 s to control the thickness. The electrolyte temperature was maintained at 25 ± 1 °C. The electroplating current density was 25 ASD (A/dm^2^). By controlling the electroplating time, copper foils of different thicknesses can be fabricated. In this study, copper foils with thicknesses ranging from 5 μm to 30 μm were electroplated to observe their variations. Cu foils with an area of 5 cm × 12 cm could be fabricated.

For each batch of copper foils, five dog-bone specimens with a 5 cm gauge length 0.3 cm gauge width were prepared for mechanical testing. The tensile properties—namely, ultimate tensile strength (UTS), yield strength (YS), and elongation—were evaluated using a universal testing machine (AGS-X, Shimadzu corp., Japan). All tests were performed in ambient air at room temperature, with a constant strain rate of 4.17 × 10^−3^ s^−1^. For microstructural analysis, a focused ion beam system (FIB, Helios G3CX, Thermo Fisher Scientific, Waltham, MA, USA) was employed, while electron backscattered diffraction (EBSD, Carl Zeiss, Oberkochen, Germany) was used to characterize grain size, crystallographic orientation, and spatial distribution within the copper films. The measurements were performed at an accelerating voltage of 20 kV with a specimen tilt of 70° and a working distance of 15 mm. A step size of 50 nm was used for all scans, and diffraction patterns were collected at a resolution of 640 × 480 pixels with a detector acquisition speed of 642.62 Hz. Grain reconstruction applied a tolerance angle of 5° and a minimum grain size threshold of 50 nm. Grain boundaries were categorized as low-angle boundaries for misorientations between 2° and 15°, and high-angle boundaries for misorientations exceeding 15°. Cross-sectional EBSD maps were used to examine the evolution of columnar grain morphology with thickness, whereas plan-view maps were used to evaluate grain size distributions.

## 3. Results and Discussion

**Figure 2:**
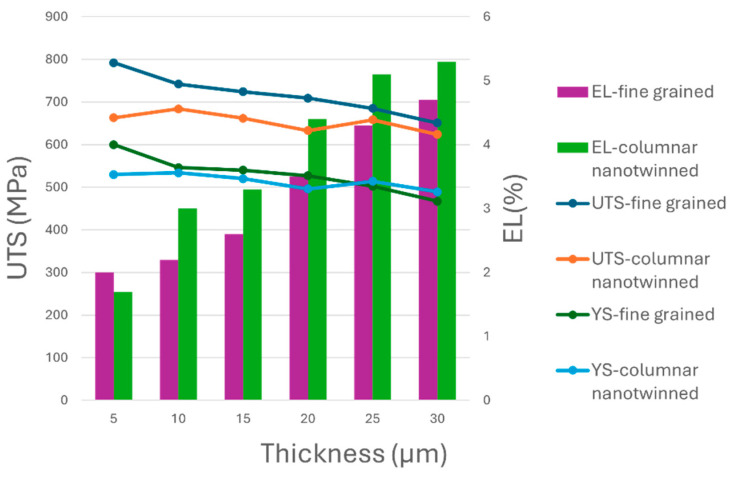
The trend of UTS, YS, and elongation with varying thickness for both FG-Cu and NT-Cu foil.

**Figure 3:**
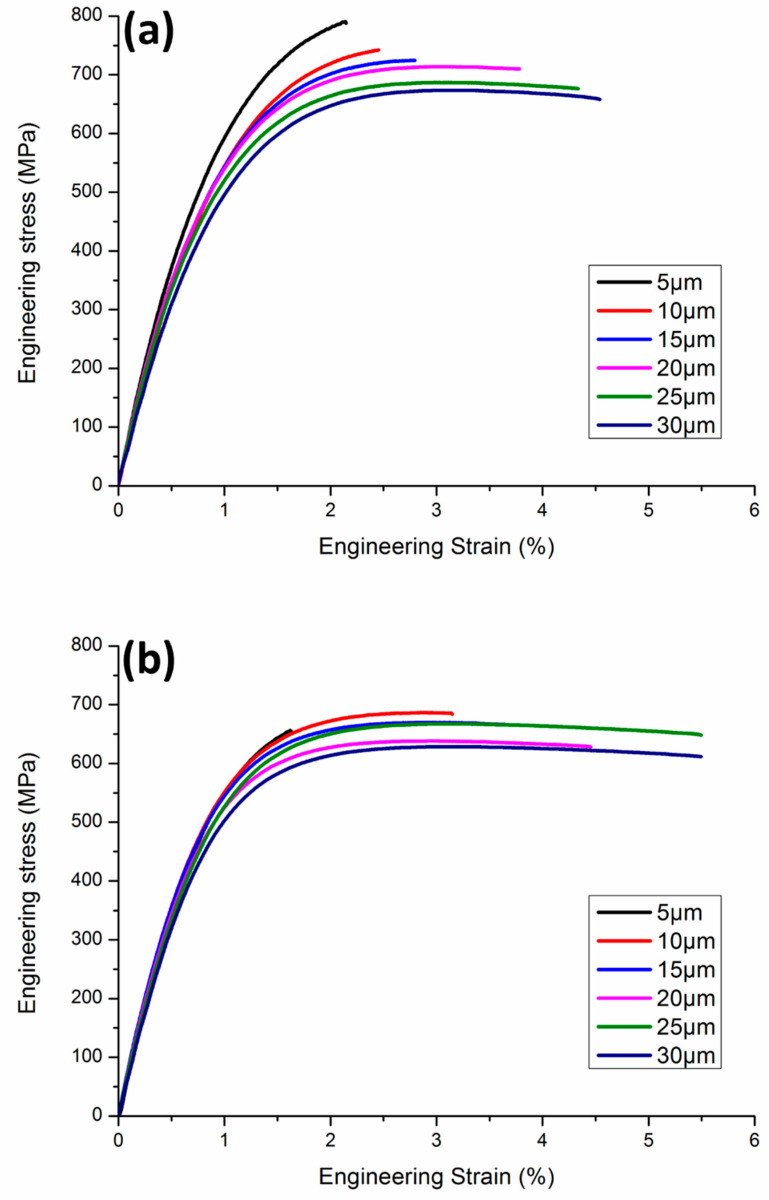
Stress-stain curve from 5 μm to 30 μm of (**a**) FG-Cu (**b**) NT-Cu.

The tensile test results are shown in Figure 2 and Figure 3. Both the FG-Cu foil and the NT-Cu foil exhibit a similar overall trend. The UTS of the 30 μm thick FG-Cu foil was measured at 651 MPa and increased with decreasing thickness, reaching 792 MPa at 5 μm. For NT-Cu foils, the UTS rose from 624 MPa at 30 μm to 663 MPa at 5 μm. The YS exhibited a similar trend. Concurrently, the elongation increases as the strength decreases. Table 1 and Table 2 show the variations in UTS, YS, and elongation with thickness for FG-Cu foil and NT-Cu foil, respectively. The changes are calculated relative to the values at a thickness of 5 μm, which is used as the reference point.

Figure 2 clearly illustrates both the magnitude and the trend of the changes. Although FG-Cu consistently exhibits higher UTS than NT-Cu across all thicknesses, Table 1 shows that the UTS of FG-Cu decreases by 17.8% from 5 μm to 30 μm. In contrast, Table 2 shows that NT-Cu shows only a 5.8% reduction over the same thickness range. As the thickness increases, the UTS values of the two types of copper foils become increasingly similar.

FG-Cu also shows a higher initial YS compared to NT-Cu at a thickness of 5 μm. However, as the thickness increases to 30 μm, the YS of FG-Cu drops by 22.1% relative to its initial value. In contrast, the YS of NT-Cu decreases by only 7.7% over the same thickness range. As shown in Figure 2, beyond 25 μm, the YS of NT-Cu surpasses that of FG-Cu at the same thickness, indicating a greater resistance of NT-Cu to thickness-induced degradation. Moreover, the gap between the two foils continues to widen with increasing thickness.

As for elongation, the performance of the two foils is relatively similar. Except at the 5 μm thickness, where NT-Cu shows slightly lower elongation than FG-Cu, NT-Cu exhibits better elongation at all other thicknesses. In summary, within the 5 to 30 μm thickness range, although FG-Cu holds a slight advantage in absolute strength values, NT-Cu demonstrates superior stability, showing less reduction in strength. This indicates that NT-Cu has a better resistance to strength degradation caused by thickness variation.

**Figure 4:**
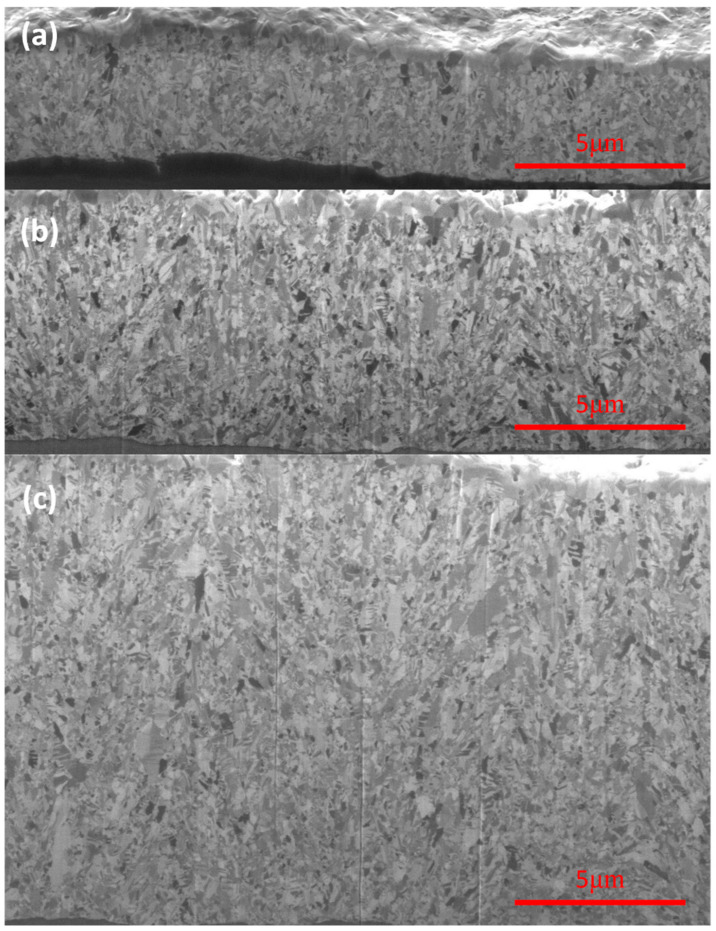
Cross-sectional ion images show the microstructure of FG-Cu foils with thickness of (**a**) 5 μm (**b**) 10 μm (**c**) 20 μm.

**Figure 5:**
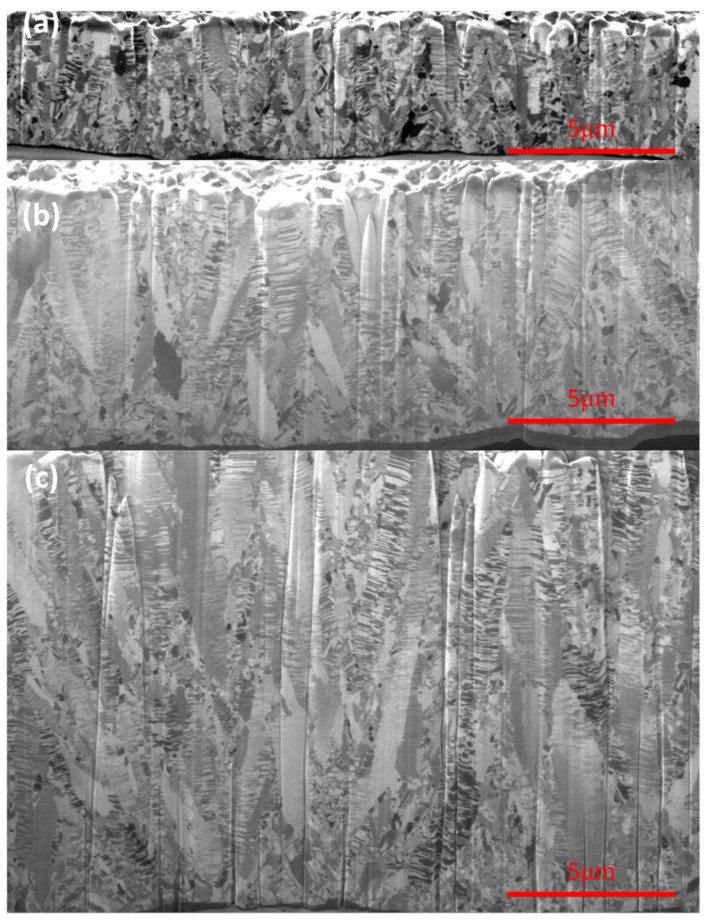
Cross-sectional ion images show the microstructure of NT-Cu foils with thickness of (**a**) 5 μm (**b**) 10 μm (**c**) 20 μm.

Figure 4 shows the cross-sectional ion images of FG-Cu foils at thicknesses of 5 μm, 10 μm, and 20 μm, respectively. The deposition direction of electroplated copper proceeds from the bottom of each image to up. Starting from Figure 4a, the 5 μm thick FG-Cu foil exhibits a fairly uniform grain size from the bottom to the top. As the plating thickness increases, Figure 4b shows that the bottom region initially deposited possesses a similar grain-size distribution to that in Figure 4a; however, with increasing thickness, the grains near the top become noticeably larger. When the thickness reaches 20 μm, as shown in Figure 4c, a greater number of large grains can be observed in the middle-to-upper region of the specimen. In FG-Cu, it is evident that as the thickness increases, locally larger grains begin to emerge during the upward electroplating process. Figure 5 shows the cross-sectional ion images of NT-Cu foils at thicknesses of 5 μm, 10 μm, and 20 μm, respectively. In contrast, Figure 5a reveals that NT-Cu already exhibits clearly visible columnar grains at a thickness of 5 μm. Except for the transition layer at the very bottom, which consists of fine grains, most of the copper foil exhibits a columnar structure. The columnar grains grow upward as the thickness increases. Unlike the FG-Cu foil, the width of the columnar grains remains nearly constant during vertical growth without significant variation. As shown by the comparison between Figure 5b and Figure 5c, even when the thickness is doubled, the columnar structure displays a similar growth distribution in the ion images. Based on rough estimations from the cross-sectional ion images, the average width of the columnar grains increases from approximately 500 nm at 5 μm thickness to about 650 nm at 20 μm. To more accurately quantify the grain size evolution with increasing thickness, we further analyzed the samples using cross-sectional EBSD.

**Figure 6:**
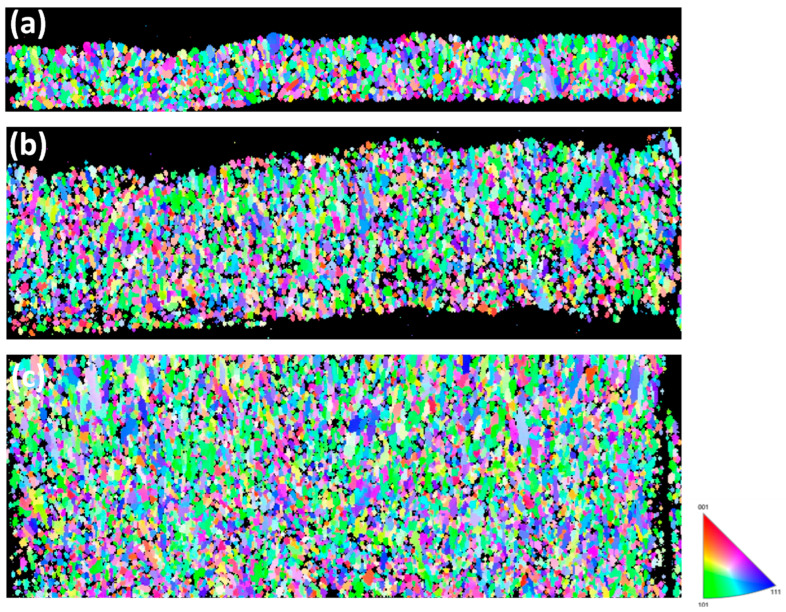
Cross-sectional EBSD images show the microstructure of FG-Cu foils with thickness of (**a**) 5 μm (**b**) 10 μm (**c**) 20 μm.

**Figure 7:**
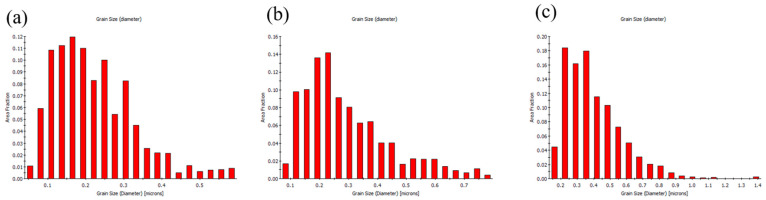
The grain size distribution of FG-Cu foils with thickness of (**a**) 5 μm (**b**) 10 μm (**c**) 20 μm.

**Figure 8:**
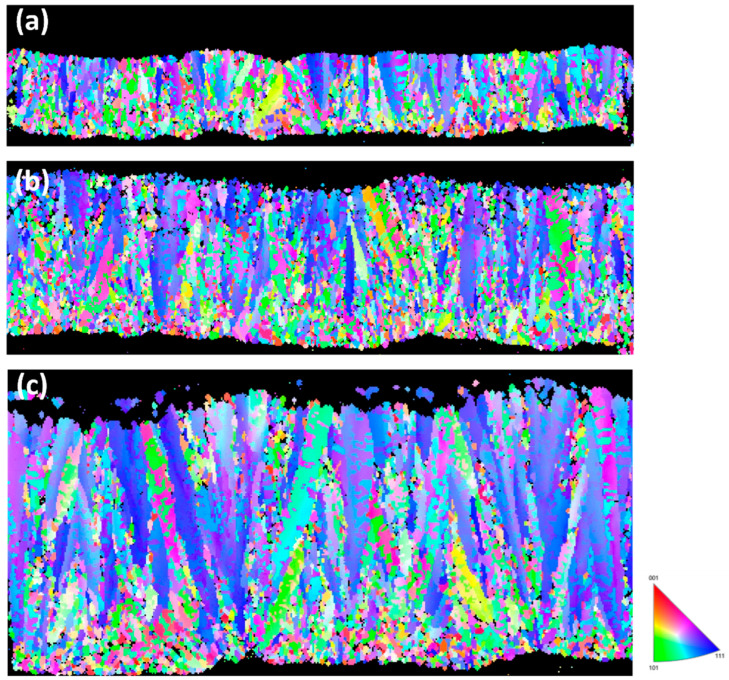
Cross-sectional EBSD images show the microstructure of NT-Cu foils with thickness of (**a**) 5 μm (**b**) 10 μm (**c**) 20 μm.

**Figure 9:**
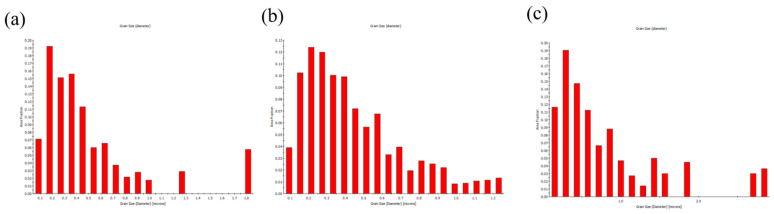
The grain size distribution of NT-Cu foils with thickness of (**a**) 5 μm (**b**) 10 μm (**c**) 20 μm.

Figure 6 presents the cross-sectional EBSD analysis of the FG-Cu foil. Since the microstructure is primarily composed of fine grains, it is difficult to visually distinguish differences among foils of various thicknesses. Figure 7 shows the corresponding grain size distributions. For the 5 μm thick foil, the grain sizes are mainly concentrated in the range of 0.1–0.2 μm. When the thickness increases to 10 μm, the peak of the distribution shifts to the range of 0.2–0.25 μm. As the thickness reaches 20 μm, the distribution primarily falls within 0.2–0.35 μm, with some grains exceeding 1 μm in size being detected. From the grain size distributions alone, it can be observed that as the thickness increases, the distribution shifts rightward along the *X*-axis, indicating a positive correlation between grain size and foil thickness. Figure 8 shows the cross-sectional EBSD analysis of the NT-Cu foil. Although some fine grains are still distributed within the structure, most of the microstructure is dominated by columnar grains. The EBSD images clearly reveal that the width of each columnar grain remains nearly constant, showing no significant increase with the elongation of the grain during growth. We also performed a statistical analysis of the grain size distribution for the NT-Cu foils. As shown in Figure 9, regardless of the foil thickness, most of the grains are distributed within the range of 0.2–0.4 μm. In contrast to the FG-Cu foils, where the grain size distribution shifts noticeably rightward along the *X*-axis with increasing thickness, the NT-Cu foils do not exhibit such a clear trend.

After analyzing the average grain size, we present the results in Table 3 and estimate the theoretical yield strength using the Hall–Petch equation [32,33]:

(1) $$\sigma_{y}=\sigma_{0}+\frac{K_{y}}{\sqrt{d}}$$

where *σ*_0_ is the friction stress of Cu; *K_y_* is the strengthening coefficient and *d* is the average grain diameter. Using FG-Cu as an example, the 5, 10, and 20 µm thick foils exhibit average grain sizes of 139, 180, and 290 nm, respectively. According to the classical Hall–Petch equation, the corresponding theoretical yield strengths are calculated to be 347, 307, and 247 MPa. However, we find that the experimentally measured yield strengths exceed the values predicted by the classical Hall–Petch relation. This discrepancy likely arises because classical formulation considers only grain-size strengthening while neglecting other contributions—such as strengthening from twin boundaries and the effects of residual/internal stresses [34]. Nevertheless, this does not alter the conclusion that, when compared against the theoretical values and grain-size dependence, our experimental results follow the same trend predicted by the Hall–Petch equation.

**Figure 10:**
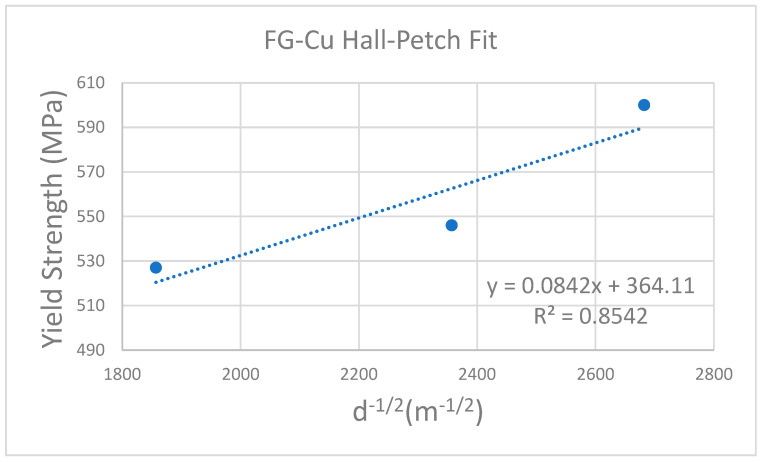

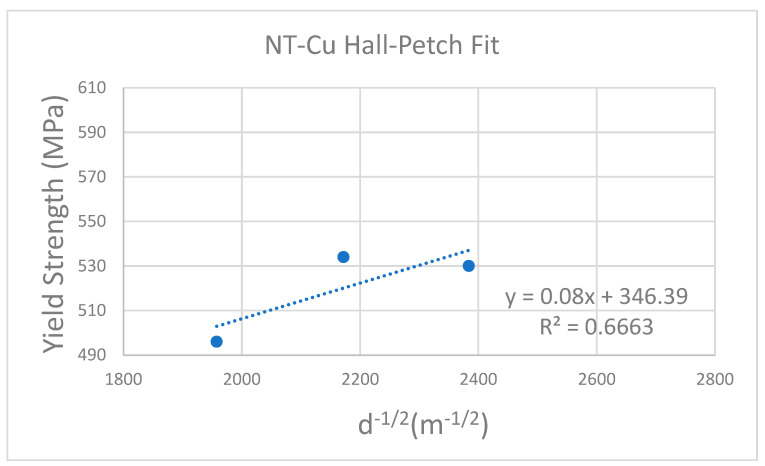
Hall-Petch fit results of FG-Cu and NT-Cu.

Using the grain sizes summarized in Table 3 and the measured yield strengths, we performed Hall–Petch fits; the results are shown in Figure 10. For FG-Cu, the fit gives R^2^ = 0.854. Although the 10 µm specimen lies slightly above the regression line, the overall trend is consistent with Hall–Petch strengthening. In contrast, the NT-Cu fit yields R^2^ = 0.666. This lower goodness-of-fit likely reflects the fact that the classical Hall–Petch relation accounts only for grain-size strengthening while neglecting other contributions—particularly those associated with the pronounced columnar grain microstructure—thereby reducing linearity for NT-Cu.

Notably, when we replace the average grain size with the average columnar grain width for NT-Cu—507, 576, 652, and 671 nm for the 5, 10, 20, and 30 µm foils, respectively—the Hall–Petch fit yields R^2^ ≈ 0.76R, indicating a more linear relationship than the fit based on average grain size. This result suggests that the columnar width (i.e., the transverse barrier spacing relevant to dislocation pile-up) is a more appropriate descriptor for NT-Cu than the cross-section EBSD grain size, and it helps explain why the mechanical properties of NT-Cu vary only weakly with thickness. It is important to note that the classical Hall–Petch relation assumes dislocation pile-up at high-angle grain boundaries and is generally valid only when the controlling length scale is the grain size [35,36]. However, for nanotwinned Cu, the dominant strengthening mechanism arises from coherent Σ3 twin boundaries. These interfaces act as highly effective barriers to dislocation transmission and confine partial dislocations, leading to a strengthening behavior that depends primarily on the twin spacing (λ). Prior studies have shown that NT-Cu follows a modified Hall–Petch-type relation, $\sigma =\sigma_{0}+k_{TB}\lambda^{-1/2}$, rather than a grain-size-based form [28,37]. Although twin spacing was not directly measured in this work, the relatively stable strength of NT-Cu with increasing thickness is consistent with the expected reduction in twin boundary density and increased twin spacing reported in the literature [38,39]. Therefore, the Hall–Petch trend observed for NT-Cu in this study should be interpreted as an effective correlation rather than a pure grain-size-controlled mechanism.

Although the primary focus of this work is the comparison between FG-Cu and NT-Cu, the thickness of the foils also plays an important mechanistic role in their deformation behavior. Several factors contribute to the observed thickness dependence. First, surface-driven grain coarsening tends to occur in thicker foils, particularly in FG-Cu, where the outer regions experience reduced growth restriction compared with the interior. As thickness increases, the effective grain size increases and the contribution of grain-boundary strengthening is reduced, consistent with the Hall–Petch trend observed in this study. Second, in very thin foils (≤10 μm), deformation is strongly affected by thin-foil dislocation mechanisms, such as limited dislocation source length, source truncation at free surfaces, and dislocation starvation. When the foil thickness approaches the characteristic slip distance, higher stresses are required for dislocation activation, leading to an elevated yield strength that cannot be explained by grain size alone. Third, geometrical constraint and plasticity size effects further strengthen ultra-thin foils. The restricted thickness suppresses multi-slip activation and favors plane-confined deformation, resulting in higher apparent strength, particularly in FG-Cu. Finally, potential substrate-free tensile testing artifacts, including slight bending, may influence the deformation response of the thinnest foils. Although polymer-backed grips and alignment procedures were used to minimize these effects, their influence cannot be entirely ruled out and are acknowledged as part of the mechanical response of ultra-thin metallic foils. These combined effects explain why thin Cu foils exhibit higher strength and why the softening trend with increasing thickness is more pronounced in FG-Cu than in NT-Cu, where twin-boundary-mediated strengthening remains dominant.

The distinct mechanical responses of FG-Cu and NT-Cu with increasing thickness can be interpreted in terms of their different deformation mechanisms. In FG-Cu, plastic deformation is dominated by grain-boundary-mediated dislocation storage and accumulation. As the foil becomes thicker, surface-driven grain coarsening reduces the density of high-angle grain boundaries, thereby lowering the number of effective barriers to dislocation motion and leading to the more pronounced softening observed in FG-Cu. The development of locally coarse grains at larger thicknesses (Figure 4 and Figure 6) further promotes strain localization and reduces yield strength.

In contrast, NT-Cu undergoes deformation that is strongly influenced by coherent Σ3 twin boundaries, which restrict dislocation slip and promote partial-dislocation transmission along the twin planes [40]. These interfaces provide highly stable and effective barriers to dislocation glide, even as the foil thickness increases. Since the columnar morphology and twin-mediated barrier spacing change only moderately with thickness (Figure 5 and Figure 8), the deformation of NT-Cu remains governed by twin-boundary interactions rather than grain-boundary hardening. As a result, NT-Cu exhibits significantly weaker thickness-dependent softening. These combined mechanisms explain why NT-Cu maintains higher strength stability across 5–30 μm, whereas FG-Cu experiences larger reductions in both UTS and YS as thickness increases.

According to the analysis results, the grain size of FG-Cu increases by 29.4% from 5 μm to 10 μm, and by 108.6% from 5 μm to 20 μm. In contrast, NT-Cu shows a more moderate grain growth, with only a 20.4% increase from 5 μm to 10 μm, and a 48.2% increase from 5 μm to 20 μm. In terms of average columnar grain width, the value increases by 13% from 5 to 10 µm, by 28% from 5 to 20 µm, and by 32% from 5 to 30 µm. Constructed on these observations, it can be reasonably inferred that the relatively lower sensitivity of NT-Cu to thickness variation stems from its distinctive columnar grain structure. This structure limits the extent of grain growth as the thickness increases, thereby reducing the loss in ultimate tensile strength (UTS) and yield strength (YS). As a result, NT-Cu can maintain more stable mechanical properties throughout the thickness variation.

## 4. Conclusions

This work provides a systematic comparison of FG-Cu and NT-Cu foils produced under identical electrodeposition conditions across a continuous 5–30 μm thickness range. Tensile testing indicated that although FG-Cu exhibited slightly higher strength value at the thinnest gauge (UTS: 792 MPa at 5 μm), its mechanical strength degraded markedly with increasing thickness, showing 17.8% and 22.1% reductions in UTS and YS, respectively, from 5 μm to 30 μm. In contrast, NT-Cu maintained outstanding thickness-dependent stability, with only 5.8% and 7.7% reductions in UTS and YS over the same range. Notably, beyond 25 μm, the yield strength of NT-Cu even surpasses that of FG-Cu, indicating enhanced resistance to thickness-induced softening. Microstructural analyses and Hall–Petch-based correlations revealed that FG-Cu undergoes pronounced grain coarsening with increasing thickness. This enlargement of grain size significantly reduces the density of high-angle boundaries that normally impede dislocation motion, thereby causing strong thickness-dependent softening. NT-Cu, on the other hand, preserves a highly stable columnar nanotwinned architecture with only moderate increases in grain or column width. Such persistence of coherent twin boundaries maintains strong barriers to dislocation motion and effectively suppresses the loss of strength with increasing thickness. These findings establish a unified thickness–microstructure–property relationship for electroplated Cu foils and provide essential mechanistic guidance for designing next-generation high-performance Cu foils for flexible electronics, advanced interconnects, lithium-ion battery current collectors, and other thickness-critical applications.

## Figures and Tables

**Table 1:** Summary of the UTS, the YS, the elongation and their respective changes for FG-Cu foils of different thicknesses.

Thickness (μm)	UTS (MPa)	Percentage Reduction (%)	YS (MPa)	Percentage Reduction (%)	Elongation (%)	Percentage Increase (%)
5	792 ± 23	-	600 ± 11	-	2 ± 0.6	-
10	742 ± 12	6.3	546 ± 10	9.0	2.2 ± 0.5	10
15	724 ± 14	8.5	540 ± 13	10.0	2.6 ± 0.6	30
20	709 ± 20	10.4	527 ± 12	12.1	3.5 ± 0.5	26
25	685 ± 20	13.5	502 ± 11	16.3	4.3 ± 0.7	75
30	651 ± 9	17.8	467 ± 10	22.1	4.7 ± 0.7	135

**Table 2:** Summary of the UTS, the YS, the elongation and their respective changes for NT-Cu foils of different thicknesses.

Thickness (μm)	UTS (MPa)	Percentage Reduction (%)	YS (MPa)	Percentage Reduction (%)	Elongation (%)	Percentage Increase (%)
5	663 ± 8	-	530 ± 10	-	1.7 ± 0.5	-
10	684 ± 8	−3.1	534 ± 12	0	3.0 ± 0.4	76
15	662 ± 7	0	520 ± 11	1.8	3.3 ± 0.5	94
20	633 ± 9	4.5	496 ± 18	6.4	4.4 ± 0.2	158
25	658 ± 6	0	514 ± 10	3	5.1 ± 0.6	200
30	624 ± 7	5.8	489 ± 10	7.7	5.3 ± 0.6	211

**Table 3:** Summary of the grainsize for both FG-Cu and NT-Cu foils of different thicknesses.

Thickness (μm)	FG-Cu Grain Size (nm)	Percentage Increase (%)	NT-Cu Grain Size (nm)	Percentage Increase (%)
5	139	-	176	-
10	180	29.4	212	20.4
20	290	108.6	261	48.2

## Data Availability

The original contributions presented in the study are included in the article, further inquiries can be directed to the corresponding author.
